# Zearalenone Mycotoxin Affects Immune Mediators, MAPK Signalling Molecules, Nuclear Receptors and Genome-Wide Gene Expression in Pig Spleen

**DOI:** 10.1371/journal.pone.0127503

**Published:** 2015-05-26

**Authors:** Gina Cecilia Pistol, Cornelia Braicu, Monica Motiu, Mihail Alexandru Gras, Daniela Eliza Marin, Mariana Stancu, Loredana Calin, Florentina Israel-Roming, Ioana Berindan-Neagoe, Ionelia Taranu

**Affiliations:** 1 Laboratory of Animal Biology, National Institute for Research and Development for Biology and Animal Nutrition, 077015, Balotesti, Ilfov, Romania; 2 Oncologic Institute Prof. Dr. I. Chiricuta, 400015, Cluj-Napoca, Romania; 3 Research Center for Functional Genomics, Biomedicine and Translational Medicine, "Iuliu Hatieganu" University of Medicine and Pharmacy, 400565, Cluj-Napoca, Romania; 4 Biotechnology Department, University of Agriculture and Veterinary Medicine, 011464, Bucharest, Romania; 5 Department of Experimental Therapeutics M.D. Anderson Cancer Center, Houston, TX, United States of America; INRS, CANADA

## Abstract

The toxicity of zearalenone (ZEA) was evaluated in swine spleen, a key organ for the innate and adaptative immune response. Weaned pigs were fed for 18 days with a control or a ZEA contaminated diet. The effect of ZEA was assessed on wide genome expression, pro- (TNF-α, IL-8, IL-6, IL-1β, IFN-γ) and anti-inflammatory (IL-10, IL-4) cytokines, other molecules involved in inflammatory processes (MMPs/TIMPs), as well as signaling molecules, (p38/JNK1/JNK2-MAPKs) and nuclear receptors (PPARγ/NFkB/AP-1/STAT3/c-JUN). Microarray analysis showed that 46% of total number of differentially expressed genes was involved in cellular signaling pathway, 13% in cytokine network and 10% in the inflammatory response. ZEA increased expression and synthesis of pro- inflammatory (TNF-α, IL-8, IL-6, IL-1β) and had no effect on IFN-γ, IL-4 and IL-10 cytokines in spleen. The inflammatory stimulation might be a consequence of JNK pathway activation rather than of p-38MAPK and NF-kB involvement whose gene and protein expression were suppressed by ZEA action. In summary, our findings indicated the role of ZEA as an immune disruptor at spleen level.

## Introduction

Zearalenone (ZEA), a macrocyclic lactone, is a mycotoxin produced by several fungi, including *Fusarium graminearum* (Gibberella zeae), *F*. *culmorum*, *F*. *cerealis*, *F*. *equiseti*, and *F*. *semitectum* [1, 2]. These fungi exists widely both in pre- and post-harvested wheat, barley, rice, maize, and other crops. Also, ZEA toxin has been detected in cereals products like flour, malt, soybeans and beer, resulting in the contamination of human foods and animal feed worldwide [3–5].

Zearalenone is known estrogenic disrupters due to its structural similarities with estrogenic receptors (ERs). The estrogenic effects of ZEA causes alterations in both laboratory (mice, rats, guinea pigs, hamsters, and rabbits) and domestic animals [6]. In human, the natural exposure to ZEA through contaminated food has been identified as the cause of female reproductive changes (breast cancer, precocious puberty) its hormonal action exceeding that of the most other naturally occurring non-steroidal estrogens [7].

After oral exposure, ZEA is rapidly absorbed, being metabolized at intestinal level and in hepatic tissue. The biotransformation of this toxin leads to generation of its metabolites, (α- and β-zearalenol, α- and β-zearalanol), all of them having biological activity. The estrogenic activity of ZEA and its metabolites is directly correlated with their binding affinity to estrogen receptors ER-α and ER-β [7]. The affinity is higher for ER-α resulting in estrogenic like effects through the activation of gene transcription via estrogen responsive elements [8]. The ERs are expressed in a large variety of tissues (uterus, mammary gland, bone, brain, and other organs), as well as on the surface of the immune system cells (ER-α on T cells, NK cells, macrophages, ER-β on B cells and monocytes) which could become a possible target for estrogenic disruptors like zearalenone [9]. Few studies reported the impact of the endocrine disrupters on the immune system [10]. However, it was demonstrated that endocrine disrupters could modulate cytokine synthesis. For example, Con-A-activated splenocytes derived from mice treated with alpha-zearalanol, a 3-4x more potent than ZEA, or with genistein, a soy isoflavone, release less interferon (IFN-γ) into culture supernatants [11] in comparison with the control.

Pigs, especially weaning piglets, are considered to be the most sensitive animal species to ZEA and its metabolites for estrogenic effects [12, 13, 8]. The most common pathological effects are decreases in fertility, anoestrus, abortion, and increased embryonic and foetal death [14]. Also, ZEA toxicity is associated with reduced litter size, changed weight of adrenal, thyroid, pituitary glands in offspring and change in serum levels of progesterone and oestradiol [8].

There are few studies regarding immunotoxic effects of ZEA in swine, especially in-depth analysis of the inflammatory processes [15–17]. It was demonstrated that ZEA and its metabolites have divergent effects on innate immunity of pig, acting as inductors [18] or suppressors [17] of the expression of pro-inflammatory cytokines in peripheral blood cells. In a recent study, we showed that ZEA contaminated diet decreased significantly the expression of pro- and anti-inflammatory markers in liver as the key organ in immune homeostasis and in the detoxification of food contaminants, and its toxicity was associated with the inhibition of several effectors of MAPKs signalłing transduction pathway [19]. In order to complete the image of the effects of ZEA on the immune defence processes we used *in vivo* genomic and proteomic approaches to evaluate the toxicity of ZEA in swine spleen as critical organ for the innate and adaptive responses to inflammation [20, 21]. Pig was also considered as human model due to the comparative sensitivity of pig and human to zearalenone.

## Materials and Methods

### Animals and treatments

Two groups of weanling piglets (TOPIGS-40 crossbred piglets 4-week-old, n = 5 per group/pen) with an initial average body weight of 9.5 ± 0.6 Kg were studied for 18 days. Animals were individually identified by ear tag. They fed on a corn-soybean meal basal diet (Table 1) and randomly assigned to either a control (diet without mycotoxin) or a ZEA contaminated group (diet contaminated with ZEA). The body weight was recorded at the beginning (day 0) and at the end of the experiment (day 18) for each animal. Feed samples collected at the beginning of the experiment were analyzed for *Fusarium* mycotoxins and nutrient content. Assigned diet and water were provided *ad libitum* every day of the experiment. In the day 18 of experiment the cumulative feed consumption was measured for each pen. They were observed twice daily and no clinical signs or death was recorded throughout the entire experimental period. At the end of the experiment, animals were stunned and slaughtered by exsanguinations in an EU-licensed abattoir according with the EU Council Directive 2010/63/CE. Organ samples were collected on ice from all animals, weighed and were stored at—80°C until the analyses. Animals were cared for in accordance with the Romanian Law 206/2004 and decision 28/2011 for handling and protection of animals used for experimental purposes. The study protocol was approved by the Ethical Committee of the National Research-Development Institute for Animal Nutrition and Biology, Balotesti, Romania.

**Table 1:** Composition of experimental diet (%).

Ingredients	Control	Contaminated diet
Wheat	15.00	15.00
Maize	53.31	53.31
Soybean meal	3.00	3.00
Sunflower meal	8.00	8.00
Powder milk	5.00	5.00
Gluten	2.00	2.00
Full fat Soybean	9.00	9.00
Salt	0.20	0.20
Monocalcium phosphate	1.30	1.30
Feed grade limestone	1.60	1.60
Methionine premix	0.10	0.10
Lysine premix	0.40	0.40
Choline premix	0.09	0.09
Vitamin mineral premix^1^	1.00	1.00
Analyzed composition		
Crude protein (g/kg)	181.7	178.8
Crude fat (g/Kg)	25.3	25.4
Mycotoxins (μg/kg)		
ZEA	40.92	316.0

^1^ Vitamin-mineral premix / kg diet: 0–24 days: 10,000 UI vit.A; 2000 vit. D; 30 UI vit. E; 2 mg vit. K; 1.96 mg vit. B_1_; 3.84 mg vit. B_2_; 14.85 mg pantothenic ac.; 19.2 mg nicotinic ac; 2.94 mg vit. B_6_; 0.98 mg folic ac.; 0.03 mg vit.B_12_; 0.06 biotin; 24.5 mg vit.C; 40.3 mg Mn; 100 mg Fe; 100 mg Cu;

### Analysis of mycotoxins

The content of ZEA in the feed was analysed by high performance liquid chromatography (HPLC) with fluorescence detection after clean-up with an immune-affinity column (Inertsil ODS-3V) and a detection limit of 6ng/g [22]. ZEA content was 40.92±0.15 ppb in the control diet, respectively 316±30.9 ppb in the zearalenone contaminated diet. Other *Fusarium* mycotoxins (DON, FB1, T-2/HT-2, OTA and AF toxins) were analysed by ELISA using a Veratox ELISA kit (Neogen, MI, 48912, USA/Canada) with a detection limit of 100, 200, 10, 1, 0.5 ppb respectively and none of them was detected.

### Microarray analysis

#### Sample collection

Animals were slaughtered at 18d and organ samples were collected on ice; spleen aliquots (30–50g) were stored at—80°C until analyzed.

#### RNA Extraction for microarray assay

Frozen spleen tissue samples (50mg) derived from pigs treated or not with ZEA were disrupted and homogenized in TRIzol Reagent (Sigma-Aldrich Chemie GmbH, Germany) using Ultra-Turrax homogenizer (IKA-Werke GmbH & Co. KG, Germany). The total RNA was extracted in chloroform-isopropanol, resuspended in 80μl of ultrapure water containing 0.02% (w/vol) dietyl pyrocarbonate (DEPC) (Sigma-Aldrich Chemie GmbH, Germany) and 1mM EDTA and treated with ribonuclease inhibitors (RNasin Plus Rnase Inhibitor, Promega Corp., USA). They were purified on silicagel columns and their quality and integrity was verified by using an Agilent 2100 bioanalyzer with the Agilent RNA 6000 nano kit (Agilent Technologies, USA). All the specimens had a RIN score 8–10. The purified RNA samples were preserved at −80°C until used.

#### Microarray assay

For the microarray analyses, Porcine (S. Scrofa) V2 Genome microarray slides with 44,000+trascripts were purchased from Agilent Technologies (Agilrom Scientific, Romania); the array consisted in 60 mer oligonucleotide probes with a total number of 45220 features and 1417 Agilent features control. The generation of microarray labelled cRNA probes (cRNA-Cy5) for each spleen sample (4 animals/treatment group) was done according to the Agilent manufacturer's protocol and using Low Input Quick Amp Labeling Kit, One-Color (48 rxn, 5190–2306). The quality of synthesised cRNA was checked by using a Nanodrop ND-1000 spectrophotometer with a minimal yield of 1.6μg and a specific activity of 6 pmol/μl Cy5/μg cRNA. Hybridisation, washing, staining and scanning followed the Agilent manufacturer's protocol. The microarray assay was performed in triplicate.

#### Microarray hybridisation

Hybridisation of microarray probes was performed on Agilent slides for Whole Porcine Genome, using *Gene Expression Hybridization Kit* (Agilent Technologies, USA), for 17 hours at 65°C. After hybridisation slides were washed in two baths with different Triton concentrations, in order to remove the unbound reagents. Slides were treated with acetonitrile and Stabilization and Drying Solution (Agilent Technologies, USA), to prevent the degradation of fluorescent dyes by atmosphere ozone. After washing steps, the slides were dried in nitrogen atmosphere and scanned.

#### Analysis of microarray

Pre-processing, normalization and differential analysis for microarray data was carried out with GeneSpring GX Version 12.6.1 software and Microsoft Excel. Low and high normalization was used to adjust the differences in intensities of the Cy5 by applying a smoothing adjustment that removes such variation. Genes significantly up and down regulated by ZEA treatment with respect to control were identified and the genes with fold change threshold greater or lower than 0.6 were considered differentially expressed. Statistical *p*-value was calculated using Student’s t-test and FDR (Benjamini Hochberg) method in order to evaluate the impact of toxin exposure. The significantly affected (*p*<0.05) gene expression profile were subjected to cluster analysis based on Pearson coefficient correlation algorithm and classified into 6 functional categories and pathways (transcription factors, growth factors, signalłing, cytokines, proliferation, inflammatory response) using GeneSpring GX Software. Microarray raw data files were submitted to ArrayExpress databank (http://www.ebi.ac.uk/arrayexpress/experiments/E-MTAB-3422/). The serial reference number for the microarray data is A-MTAB-556.

#### Microarray validation

Microarray results were validated using qPCR, for the following genes: TGF β-2 (transforming growth factor β-2), CXCL2 (chemokine (C-X-C motif) ligand 2), TLR-7 (Toll-like Receptor-7) and *FOXP3* (forkhead box P3). Primer pairs used for validation are listed in Table 2. The qPCR was performed as described below.

**Table 2:** Nucleotide sequences of primers for microarray validation and for Real-Time PCR.

Gene	Accesion no.	Primer source	Primer sequence (5`→3`)	Orientation	Tm (°C)	Amplicon (bp)	References
**TGFβ2**	XM_005653762.1	Pig	CGATGATGATGTTGATGATGG	forward	55	69	[23]
			GCAAGGCTTTCTTGTATTTTCTTG	reverse	58		
**CXCL2**	NM_001001861.2	Pig	CCGTGCAAGGAATTCACCTC	forward	60	125	[24]
			TGCGGGGTTGAGACAAACTT	reverse	60		
**FoxP3**	NM_001128438.1	Pig	TTCCCAGACTTTCTTTCACAACAT	forward	59	113	[25]
			GCTGCTTCTCTGGAGCCTCCAG	reverse	65		
**TLR7**	NM_001097434.1	Pig	CCAACAACCGGCTTGATTTAC	forward	58	100	[26]
			TCTGATTGAAAATAGTGGCTGTTACTACT	reverse	61		
**TNF-α**	NM_214022	Pig	ACTGCACTTCGAGGTTATCGG	forward	60	118	[27]
			GGCGACGGGCTTATCTGA	reverse	60		
**IL-8**	NM_213867.1	Pig	GCTCTCTGTGAGGCTGCAGTTC	forward	58	79	[27]
			AAGGTGTGGAATGCGTATTTATGC	reverse	54		
**IL-6**	NM_214399	Pig	GGCAAAAGGGAAAGAATCCAG	forward	57	87	[27]
			CGTTCTGTGACTGCAGCTTATCC	reverse	61		
**IL-1β**	NM_214055	Pig	ATGCTGAAGGCTCTCCACCTC	forward	62	89	[28]
			TTGTTGCTATCATCTCCTTGCAC	reverse	59		
**IL-10**	NM_214041.1	Pig	GGCCCAGTGAAGAGTTTCTTTC	forward	54	51	[27]
			CAACAAGTCGCCCATCTGGT	reverse	55		
**IFN-γ**	NM_213948.1	Pig	TGGTAGCTCTGGGAAACTGAATG	forward	54	79	[29]
			GGCTTTGCGCTGGATCTG	reverse	55		
**IL-4**	NM_214123.1	Pig	CAACCCTGGTCTGCTTACTG	forward	52	173	[30]
			CTTCTCCGTCGTGTTCTCTG	reverse	52		
**MMP-2**	NM_214192.1	Pig	GGCTTGTCACGTGGTGTCACT	forward	57	68	[31]
			ATCCGCGGCGAGATCTTCT	reverse	55		
**MMP-9**	NM_001038004.1	Pig	GAAGCTTTAGAGCCGGTTCCA	forward	55	96	[31]
			GGCAGCTGGCAGAGGAATATC	reverse	55		
**TIMP-1**	NM_213857.1	Pig	CAAAACTGCAGGTGGTGATGTG	forward	55	70	[31]
			CGCAGCCAGGAGTTTCTCAT	reverse	55		
**TIMP-2**	NM_001145985.1	Pig	CAGGTACCAGATGGGCTGTGA	forward	56	77	[31]
			ACTCGTCCGGAGAGGAGATGTAG	reverse	57		
**PPARγ**	NM_214379.1	Pig	ACTGTCGGTTTCAGAAGTGC	forward	53	138	[32]
			CAGCAGACTCTGGGTTCAGT	reverse	53		
**p38α**	XM_003356616.1	Pig	TGCAAGGTCTCTGGAGGAAT	forward	52	109	[33]
			CTGAACGTGGTCATCCGTAA	reverse	52		
**JNK1**	XM_003359272.1	Pig	TGCTTTGTGGAATCAAGCAC	forward	51	60	[33]
			TGGGCTTTAAGTCCCGATG	reverse	51		
**JNK2**	XM_003354171.2	Pig	TATTATCGGGCACCAGAAGTC	forward	51	97	[33]
			AACCTTTCACCAGCTCTCTCA	reverse	53		
**NFkB1/p50**	NM_001048232.1	Pig	TCGCTGCCAAAGAAGGACAT	forward	54	101	[34]
			AGCGTTCAGACCTTCACCGT	reverse	56		
**NFkB/p65**	NM_001114281.1	Pig	CGAGAGGAGCACGGATACCA	forward	55	62	[34]
			GCCCCGTGTAGCCATTGA	reverse	54		
**STAT3**	XM_005668829.1	Pig	AACTCCTAGGACCTGGTGTGAA	forward	50	193	[35]
			CGCTCCCTCTCCTTACTGATAA	reverse	50		
**AP1**	XM_005659091.1	Pig	CCCAAGATCCTGAAGCAGAG	forward	62	136	[36]
			GATGTGCCCGTTACTGGACT	reverse	62		
**c-JUN**	NM_213880.1	Pig	GAAAAGGAAGCTGGAGAGGAT	forward	57	172	[33]
			CTGCTGCGTTAGCATGAGTT	reverse	59		
**Cyclophilin A**	NM_214353.1	Pig	CCCACCGTCTTCTTCGACAT	forward	54	92	[37]
			TCTGCTGTCTTTGGAACTTTGTCT	reverse	55		
**β-actin**	NM_213978.1	Pig	GGACTTCGAGCAGGAGATGG	forward	60	230	[38]
			GCACCGTGTTTGCGTAGAGG	reverse	62		

### Evaluation of genes involved in inflammatory responses by qPCR

#### Extraction of total RNA and cDNA synthesis

Tissue samples were taken from the spleen and stored at -80°C until RNA extraction. 100 mg of spleen samples were disrupted and homogenized in RTL buffer (QIAGEN GmbH, Germany) using Ultra-Turrax homogenizer (IKA-Werke GmbH & Co. KG, Germany). Total RNA was extracted using Qiagen RNeasy midi kit (QIAGEN GmbH, Germany), according to the manufacturer’s recommendations and extracted RNA was further treated with a ribonuclease inhibitor (RNasin Plus RNase Inhibitor; Promega Corp., USA). A Nanodrop ND-1000 spectrophotometer (Thermo Fischer Scientific, USA) was used to deteremine the quantity and quality of extracted total RNA. The integrity of RNA was verified by agarose gel electrophoresis. The total RNA isolated from each sample was further used to generate cDNA using M-MuLV Reverse Trascriptase kit (Fermentas, Thermo Fischer Scientific, USA) according to the manufacturer’s protocol [19].

#### Quantitative Real-Time PCR

Fluorescent real-time PCR was carried out to analyze genes important for inflammatory responses, such as pro-inflammatory (IL-1β, TNF-α, IL-6, IL-8 and IFNγ) and anti-inflammatory (IL-4 and IL-10) cytokines, MMPs (MMP-2 and -9) and their natural inhibitors TIMPs (TIMP-1 and -2), MAPKs (p38MAPK, JNK1/JNK2) and nuclear receptors (PPAR-γ, NFkB1/p50, NFkB/p65, AP1, STAT3 and c-JUN). Reactions were set up in a total volume of 20μl using 5μl of cDNA (diluted 1:10 with nuclease-free water), Maxima SYBR Green/Fluorescein qPCR Master Mix 2X (Fermentas, Thermo Fischer Scientific, USA), 0.3μM each of gene-specific primer. The primer pairs used in the present study, listed in Table 2, were obtained from Eurogentec (San Diego, USA). The qPCR was performed using the Rotor-Gene-Q (QIAGEN GmbH, Germany) machine and the cycling conditions were: 50°C for 2 min, 95°C for 15s, followed by 40 cycles of 95°C for 15s, 60°C for 15s and 72°C for 15s with a single fluorescence measurement; a final elongation step was carried out at 72°C for 10 min. All samples were measured in duplicate and the specificity of the PCR products was confirmed by analysis of the melting curve (50°C–95°C). Negative controls were used for each primer pair, which consisted in all of the components of the qPCR mix except cDNA. The relative quantification of gene expression changes were quantified using the comparative method [39–41]. The expression levels of two reference genes, Cyclophilin A and β -actin were used for data normalisation. These reference genes were experimentally validated for spleen tissues and the lack of treatment effect and expression variation was the criteria for reference gene choice. The results were expressed as relative fold change (Fc) in comparison with control samples.

### Measurement of cytokine production

500 mg of spleen samples were homogenized in phosphate buffer containing 1% igepal, 0.5% sodium deoxycholate, 0.1% SDS and complete (EDTA-free) protease inhibitor cocktail tablets, using Ultra-Turrax homogenizer (IKA-Werke GmbH & Co. KG, Germany). The lysates were kept 30 min on ice, and then centrifuged at 10,000 g at 4°C for 10 min. Supernatants were analyzed for protein content using commercial kit (Pierce BCA Protein Assay Kit, Thermo Fischer Scientific, USA) and cytokine concentrations in the supernatants were determined by ELISA, using the commercially available kits (R&D Systems, Minneapolis, MN 55413, USA), according to the manufacturer’s instructions [17]. Optical densities were measured on an ELISA reader (Tecan, Sunrise, Austria) at a wavelength of 450 nm. Dilution of recombinant swine IL-1β, TNF-α, IL-4, IL-6, IL-8, IL-10 and IFNγ were used as standards, and data were analyzed against the linear portion of the generated standard curve. Results were expressed as picograms of cytokine/mL and they were normalised to total protein concentration.

### Measurement of MMP-2 and MMP-9 gelatinase activity

Frozen spleen samples of 50 mg were homogenized for 30 minutes on ice in TBS lysis buffer (50 mM Tris Buffered Saline pH 7.5, 250 mM NaCl, 10% glycerol, 1% Triton-X 100 and complete EDTA-free protease inhibitor cocktail tablets). The homogenates were clarified by centrifugation at 1200 rpm for 10 minutes at +4°C and the supernatants were re-centrifuged at 15000 rpm for 15 minutes at +4°C. The final supernatants were analyzed for total protein content, using a commercial kit (Pierce BCA Protein Assay Kit, Thermo Fischer Scientific, Rockford, USA) and the aliquots were frozen at—80°C until processing. The determination of gelatinase activity of MMP-2 and MMP-9 was assessed by zymography, using SDS-PAGE electrophoresis in the presence of 0.1% gelatin [42]. After electrophoresis, the proteins were renaturated by incubation of gels in 2.5% Triton-X100 solution for 30 minutes. The activity of gelatinase was developed by incubation of the gel, at 37°C for 18 hours, in an enzymatic substrate (50 mM Tris, pH 7.4, with 5 mM CaCl_2_ and 0.2 mM NaCl_2_). After gel staining with Coomassie Brilliant Blue was visualized the gelatinolytic activity as a clear lane on a blue background. The obtained zymograms were scanned and enzymatic activities of MMPs were quantified using GelQuant software (DNR Bio-Imaging Systems LTD, Jerusalem, Israel). All densitometry results were expressed as arbitrary units (AU).

### Detection of phospho-p38 and JNK1 MAPKs and of NFkB/p56 by western blotting

#### Preparation of cytoplasmic and nuclear extracts for western blot

100 mg of spleen tissue samples were used for extraction of cytoplasmic and nuclear fractions. Cytoplasmic—nuclear fractionation was conducted using the NE-PER Nuclear and Cytoplasmic Extraction Reagents kit (Thermo Fisher Scientific, Rockford, USA) according to the manufacturer’s protocol. The resulted cytoplasmic and nuclear extracts were analyzed for total protein content, using a commercial kit (Pierce BCA Protein Assay Kit, Thermo Fischer Scientific, Rockford, USA) and the aliquots were frozen at—80°C until processing.

#### Western blotting assays

Western blotting assays were used to analyze the expression of phospho-p38 MAPK and phospho-NFkB in the spleen tissues. Samples of cytoplasmic and nuclear extracts (25 μg) were diluted (4:1) in Laemli denaturating buffer (Bio-Rad, California, USA), boiled, subjected to 10% sodium dodecylsulfate—polyacrylamide gel electrophoresis (SDS-PAGE), and electro-transferred to a nitrocellulose membrane. Equal protein loading and transfer was verified visually by staining membranes with Ponceau red solution. The membrane was then blocked with 5% non-fat dry milk in Tris Buffered Saline (pH 7.5) with 0.1% Tween 20. The phospho-p38MAPK, JNK1 and phospho-NFkB/p65 proteins were detected by incubation with their respective primary antibodies. Rabbit anti-β-actin antibody was used as control. The following step was membrane incubation with a horseradish peroxidase-conjugated goat anti-rabbit antibody, diluted 1:2000 (Cell Signaling Technology, Danvers, MA, USA). The immunocomplexes were visualized by the ECL chemiluminescent method using Clarity Western ECL Substrate (Bio-Rad, California, USA). Immunoblotting images were visualized using MicroChemi Imager (DNR Bio-Imaging Systems LTD, Jerusalem, Israel). The level of proteins was evaluated using GelQuant software (DNR Bio-Imaging Systems LTD, Jerusalem, Israel). The results were expressed as ratio between the level of phospho-p38 MAP kinase, JNK1, phospho- NFkB/p65 and the expression level of β-actin.

### Statistical analysis

All data are expressed as mean ± standard error of the mean (SEM). ANOVA & t-test analysis was performed to investigate the statistical differences between groups for all parameters analysed. Further differences between means were determined by the least square difference Fisher procedure. The correlations between analysed genes and proteins were computed using R software (http://www.r-project.org/). Values of *p*<0.05 were considered significant.

## Results

### Microarray analysis

A porcine genome microarray was used in this study to investigate the differential gene expression profiles in spleen tissues derived from pigs fed diet contaminated or not with zearalenone for 18 days. According to GeneSpring GX Version 12.6.1 Software, out of 40,000 genes on the chip, 480 genes were differentially (288 genes up-regulated and 192 genes down-regulated) expressed in spleen isolated from pigs fed ZEA diet in comparison with control animals.

### Functional classification of differentially expressed genes

**Fig 1:**
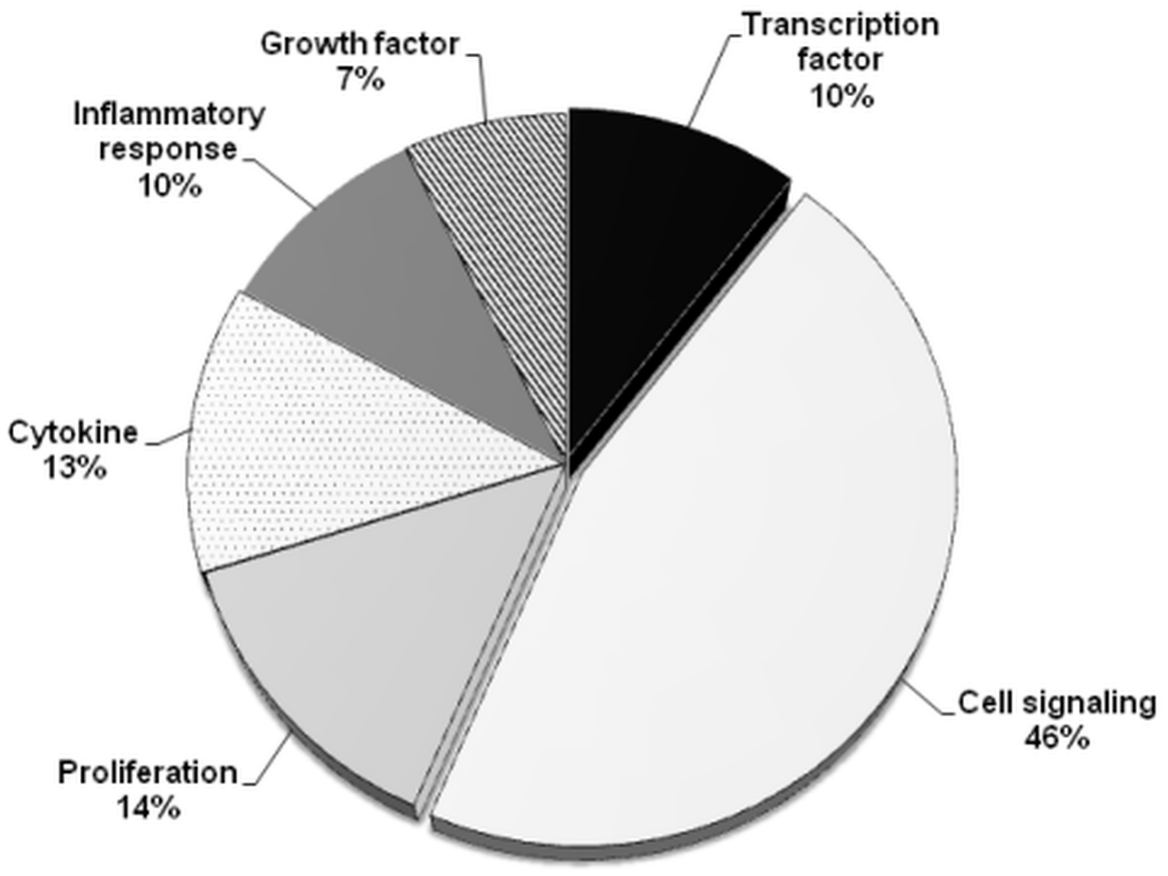
Effect of ZEA diet on global gene profile in spleen. Functional cluster analyses (%). Spleen responsive genes were categorised in 6 functional groups based on gene ontology available in the Entrez data base and using GeneSpring GX Version 12.6.1 software and Microsoft Excel.

**Fig 2:**
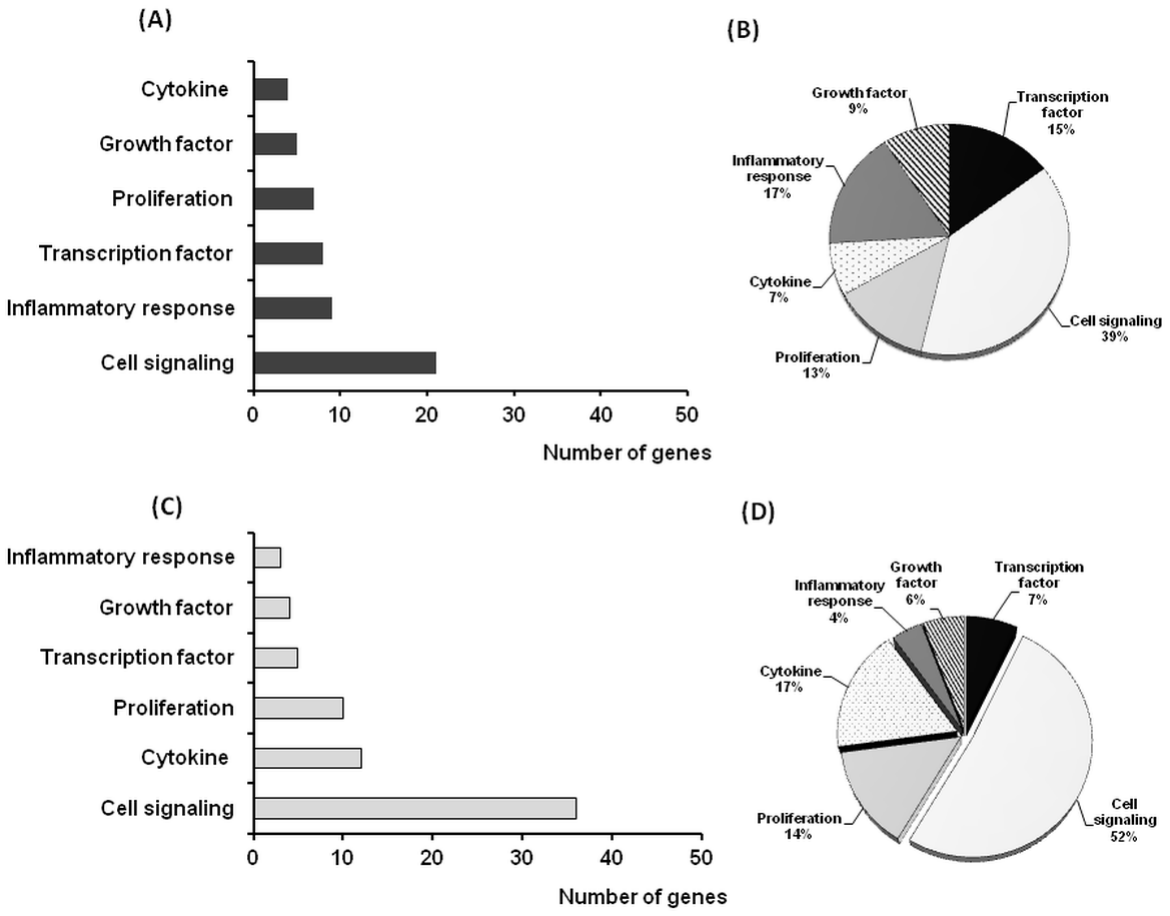
Functional cluster analyses. Differentially regulated gene profile in spleen. Spleen up- and down-regulated genes of the 6 functional groups based on gene ontology available in the Entrez data base. (A) Number of up-regulated genes. (B) % of up-regulated genes. (C) Number of down-regulated genes. (D) % of down-regulated genes.

Statistically differentiated genes belonging to specific functional clusters and pathways were identified using GeneSpring GX Version 12.6.1 Software. Analysis showed that, among genes affected by ZEA treatment, a total number of 57 genes are involved in cellular signalłing pathway, representing 46% from the total number of differentially expressed genes (Fig 1). Data presented in Fig 2 and Table 3 showed that of these, 21 genes were up-regulated (with the most increased fold change for OR10J3 gene, 13.55, *p* = 0.011), and 36 genes were down-regulated (the most affected gene being RXFP1, 0.25 Fc, *p* = 0.001).

**Table 3:** Microarray analysis showing 60 differentially expressed genes in spleen isolated from pigs fed with ZEA diet.

Gene symbol	Gene description	ZEA (Fc)	*p*-value	Regulation
	**Cell signaling**			
AFAP1L2	actin filament associated protein 1-like 2	2.46	0.025	Up
ARHGEF11	Rho guanine nucleotide exchange factor (GEF) 11	2.01	0.005	Up
OR10J3	olfactory receptor, family 10, subfamily J, member 3	13.55	0.011	Up
NXPH3	neurexophilin 3	6.50	0.000	Up
RXRG	Retinoid X receptor, gamma	2.16	0.011	Up
TNNC2	troponin C type 2 (fast)	9.13	0.001	Up
PCSK6	proprotein convertase subtilisin/kexin type 6	2.66	0.019	Up
JAG1	jagged 1	2.43	0.000	Up
KRT8	keratin 8	2.16	0.032	Up
S100A12	S100 calcium binding protein A12	2.28	0.027	Up
TGFB2	transforming growth factor, beta 2	2.00	0.006	Up
PTGDR2	prostaglandin D2 receptor 2	2.14	0.032	Up
GPR128	G protein-coupled receptor 128	0.49	0.002	Down
CCRL1	chemokine (C-C motif) receptor-like 1	0.48	0.000	Down
OR6C68	olfactory receptor, family 6, subfamily C, member 68	0.34	0.006	Down
RXFP1	relaxin/insulin-like family peptide receptor 1	0.25	0.001	Down
OMG	oligodendrocyte myelin glycoprotein	0.43	0.014	Down
OMG	oligodendrocyte myelin glycoprotein	0.46	0.030	Down
LPL	lipoprotein lipase	0.43	0.004	Down
CA2	carbonic anhydrase II	0.42	0.018	Down
OMG	oligodendrocyte myelin glycoprotein	0.42	0.006	Down
	**Cellular proliferation**			
PRG4	Proteoglycan 4	2.19	0.029	Up
SLC11A1	solute carrier family 11 (proton-coupled divalent metal ion transporters), member 1	2.38	0.042	Up
JAG1	jagged 1	2.43	0.000	Up
TGFB2	transforming growth factor, beta 2	2.00	0.006	Up
EPO	erythropoietin	0.35	0.004	Down
E2F1	E2F transcription factor 1	0.45	0.024	Down
FOXP3	forkhead box P3	0.31	0.029	Down
CD40LG	CD40 ligand	0.47	0.018	Down
	**Cytokines**			
SLC11A1	solute carrier family 11 (proton-coupled divalent metal ion transporters), member 1	2.38	0.042	Up
TGFB2	transforming growth factor, beta 2	2.00	0.006	Up
AFAP1L2	actin filament associated protein 1-like 2	2.46	0.025	Up
PRG4	Proteoglycan 4	2.19	0.029	Up
CD40LG	CD40 ligand	0.47	0.018	Down
EPO	erythropoietin	0.35	0.004	Down
CD40LG	CD40 ligand	0.47	0.018	Down
TLR7	toll-like receptor 7	0.40	0.035	Down
	**Inflammatory responses**			
SLC7A2	solute carrier family 7 (cationic amino acid transporter, y+ system), member 2	3.03	0.004	Up
AFAP1L2	actin filament associated protein 1-like 2	2.46	0.025	Up
SLC11A1	solute carrier family 11 (proton-coupled divalent metal ion transporters), member 1	2.38	0.042	Up
SERPINA3	serpin peptidase inhibitor, clade A (alpha-1 antiproteinase, antitrypsin), member 3	3.39	0.001	Up
CXCL2	chemokine (C-X-C motif) ligand 2	2.43	0.011	Up
S100A12	S100 calcium binding protein A12	2.28	0.027	Up
FOXP3	forkhead box P3	0.31	0.029	Down
CD40LG	CD40 ligand	0.47	0.018	Down
TLR7	toll-like receptor 7	0.40	0.035	Down
	**Growth factors**			
AFAP1L2	actin filament associated protein 1-like 2	2.46	0.025	Up
PCSK6	proprotein convertase subtilisin/kexin type 6	2.66	0.019	Up
JAG1	jagged 1	2.43	0.000	Up
TGFB2	transforming growth factor, beta 2	2.00	0.006	Up
FOXP3	forkhead box P3	0.31	0.029	Down
	**Transcription factors**			
ID2	inhibitor of DNA binding 2, dominant negative helix-loop-helix protein	1.93	0.018	Up
MEOX2	mesenchyme homeobox 2	2.38	0.027	Up
MITF	microphthalmia-associated transcription factor	2.04	0.021	Up
ID2	inhibitor of DNA binding 2, dominant negative helix-loop-helix protein	1.95	0.003	Up
NFIX	Nuclear factor 1 X-type	2.14	0.009	Up
HDAC11	histone deacetylase 11	25.99	0.001	Up
E2F1	E2F transcription factor 1	0.45	0.025	Down
FOXP3	forkhead box P3	0.31	0.029	Down
RAB18	RAB18, member RAS oncogene family	0.49	0.023	Down

ZEA treatment affected also genes related to proliferation and cytokine (14% and 13% from total altered genes, Fig 1). For cellular proliferation process, a number of 7 genes were up-regulated, (e.g. JAG1 gene, 2.43 fold increase, *p*<0.001) and 10 genes were down-regulated (FoxP3 gene, 0.31 Fc, *p* = 0.029) due to the effect of ZEA diet (Table 3). Expression of 12 genes belonging to cytokine pathway are down-regulated (Fig 2), TLR7 gene being the most down-regulated (0.40 Fc, *p* = 0.035, Table 3).

Expression of gene sets related to inflammatory response and transcription factor pathways are affected by ZEA diet in proportion of 10% of the total significantly affected genes. As shown in Fig 2, the tendency of ZEA treatment was to up-regulate genes involved in inflammatory responses (9 genes up-regulates, with notable up-regulation of SERPINA3 gene, 3.39 Fc, *p* = 0.001, Table 3) as well as in transcription factor pathway (8 genes affected, the most up-regulated being HDAC11, 25.99 Fc, *p* = 0.001, Table 3).

### Validation of differentially expressed genes by qPCR

**Fig 3:**
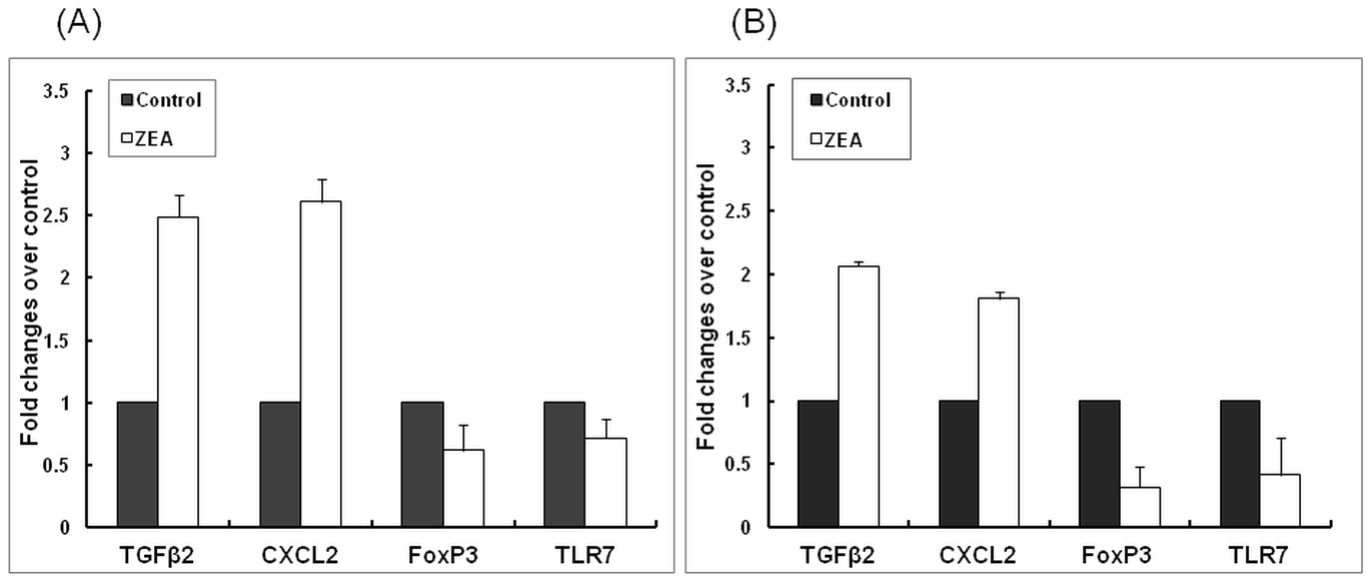
Validation of microarray results by real-time RT-PCR. Gene expression of 4 selected genes (TGFb2, CXCL2, FoxP3, TLR7) obtained by qPCR (A) was compared with that obtained by microarray (B). Results are expressed as average and SEM.

The expression level of 4 genes, TGFβ-2 (transforming growth factor β-2), CXCL2 (chemokine (C-X-C motif) ligand 2), TLR-7 (Toll-like Receptor-7) and FOXP3 (forkhead box P3) were analysed by quantitative Real-Time PCR (qPCR) in order to validate the microarray results. The expression level of genes found to be down-regulated in microarray analysis (FoxP3, 0.31 Fc, *p* = 0.029 and TLR7, 0.40 Fc, *p* = 0.035, Table 3, Fig 3B) showed the same pattern in qPCR analysis (0.62 Fc, *p* = 0.161 for FoxP3 and 0.72 Fc, *p* = 0.486 for TLR7, Fig 3A). Also, TGFβ-2 and CXCL2 were found to be up-regulated both in microarray analysis (2.00 Fc for TGFβ-2, *p* = 0.006 and 2.43 Fc for CXCL2, *p* = 0.011, Table 3, Fig 3B) as well as in qPCR (2.49 Fc, *p* = 0.095 for TGFβ-2 gene and 2.61 Fc, *p* = 0.080 for CXCL2 gene, Fig 3A).

### The influence of ZEA diet on pro- and anti-inflammatory cytokines gene expression and protein concentration in pig spleen tissues

**Fig 4:**
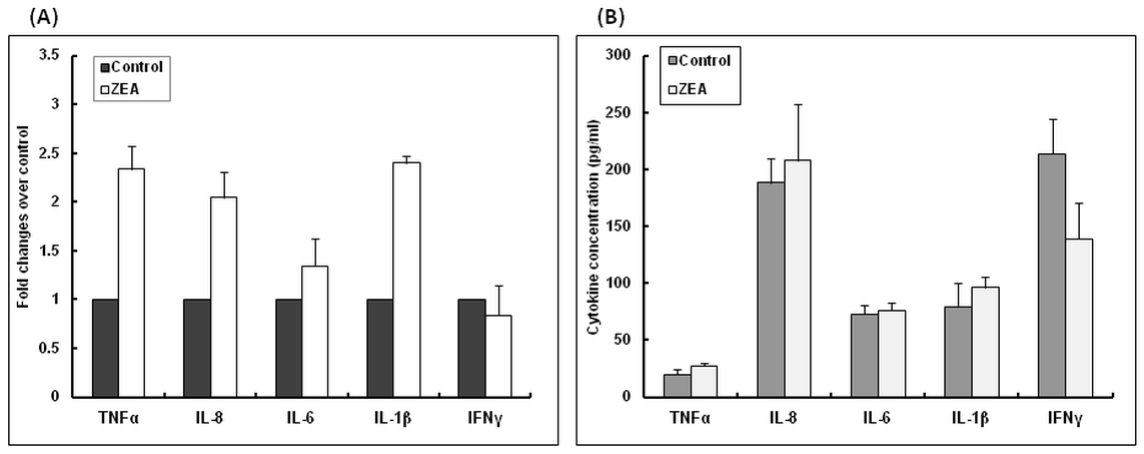
**A.** Effect of ZEA diet on pro-inflammatory cytokines expression in spleen. Gene expression of pro-inflammatory cytokines TNF-α, IL-8, IL-6, IL-1β and IFN-γ in spleen samples derived from animals treated *in vivo* with ZEA and analysed by qPCR. Results are expressed as fold changes after normalization of the expression of target cytokine gene to the mean of 2 internal reference genes. Values are the means ± SEM, from two independent replicates spleen samples/group (n = 5). Statistical analysis was performed using one-way ANOVA followed by Fisher test (* = *P*< 0.05, ZEA-contaminated spleen versusControl spleen). **B.** Effect of ZEA diet on pro-inflammatory cytokines concentration in spleen. Effect of ZEA contaminated diet on TNF-α, IL-8, IL-6, IL-1β and IFN-γ synthesis in the spleen. Spleen supernatants were analysed for cytokine protein concentration using ELISA commercial kits and the manufacturer’s instructions. Optical densities were measured at 450 nm. Values are the means ± SEM, from two independent replicates/spleen samples/group (n = 5). Statistical analysis was performed using one-way ANOVA followed by Fisher test.

Microarray results presented in previous section showed that dietary ZEA significantly altered the genes involved in the cytokine pathway and inflammatory response. In the next step, additional analyses were performed in order to evaluate the capacity of ZEA to modulate *in vivo* pro- and anti-inflammatory cytokine network in swine spleen both at mRNA and protein level. As can be observed in Fig 4A, ZEA contaminated diet induced at spleen level an increase of several pro-inflammatory cytokines gene expression. This up-regulation of cytokines mRNA was observed for TNF-α, with an increase of 133.67%, (*p*<0.05); IL-8: 104.70% (*p*<0.05); IL-6: 34.49% (*p*>0.05) and IL-1β: 139.91% (*p*>0.05) when compared with control group. No effect of ZEA on IFNγ (Fig 4A) and anti-inflammatory cytokines expression IL-4 and IL-10 (Table 4) was obtained. As expected an increase tendency or no effect was also observed for these cytokines at protein level (Fig 4B, Table 5).

**Table 4:** Effect of *in vivo* exposure of piglets to ZEA on anti-inflammatory cytokine gene expressions in spleen tissue.

Cytokine gene expression (Fc)^b^	Experimental groups^a^		ZEA effect^c^
	Control	ZEA contamination	*P*-value
**IL-10**	1	1.07 ± 0.37	NS
**IL-4**	1	1.05 ± 0.22	NS

^a^Pigs were fed for 18 days with a control diet or a diet contaminated with ZEA. At the end of the experiment, spleen samples from all animals (n = 5) were collected and analyzed for cytokine mRNA expression by quantitative Real-Time PCR.

^b^results are expressed as fold changes (Fc) after normalization of the expression of target cytokine gene to the arithmetic mean of 2 internally expressed reference genes (mean ± SEM).

^c^Anova-one way followed by Ficher tests were realized to analyze the effect of ZEA treatment on cytokine mRNA expression. Values of *P* < 0.05 were considered significant, NS = not significant.

**Table 5:** Effect of *in vivo* exposure of piglets to ZEA on anti-inflammatory cytokine protein level in spleen tissue.

Cytokine concentration (pg/ml)^b^	Experimental groups^a^		ZEA effect^c^
	Control	ZEA contamination	*P*-value
**IL-10**	11.77 ± 1.04	11.25 ± 0.45	NS
**IL-4**	22.64 ± 1.39	19.83 ± 1.47	NS

^a^Pigs were fed for 18 days with a control diet or a diet contaminated with ZEA. At the end of the experiment, spleen samples from all animals (n = 5 per group) were collected and analyzed for cytokine level by ELISA.

^b^results are expressed as concentration in pg/ml (mean ± SEM).

^c^Anova-one way followed by Ficher tests were realized to analyze the effect of ZEA treatment on cytokine production. Values of *P* < 0.05 were considered significant, NS = not significant.

### The influence of ZEA diet on MMPs and TIMPs gene expression and on MMPs activity in swine spleen tissues

**Fig 5:**
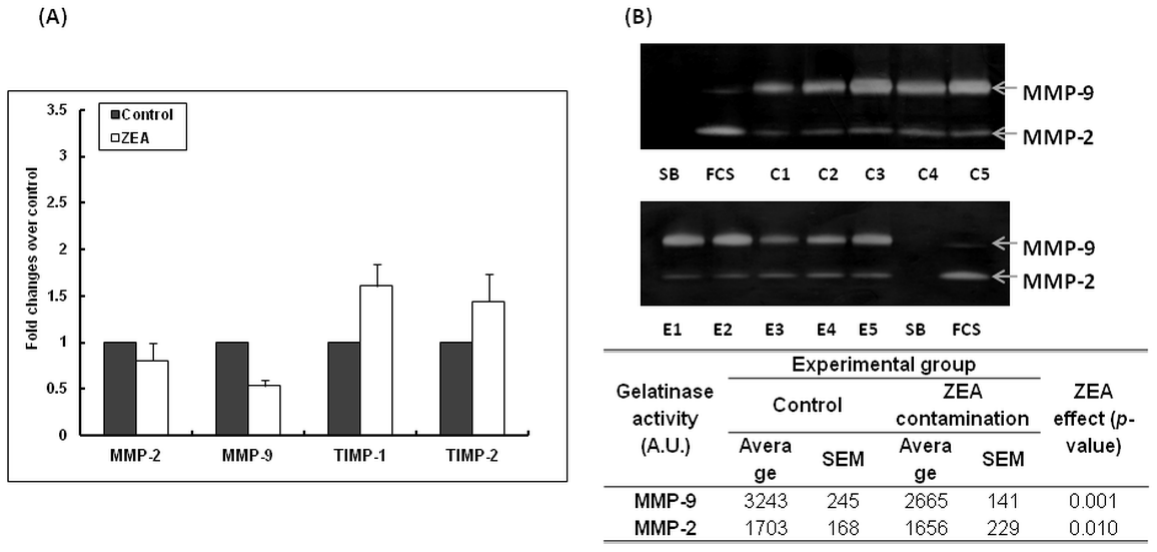
**A.** Effect of ZEA diet on the gene expression of pro-inflammatory matrix metalloproteinases (MMP) and tissue inhibitors of matrix metalloproteinases (TIMP) in the spleen. Gene expression of MMP/TIMP analysed by qPCR. Results are expressed as fold changes after normalization of the expression of target MMP/TIMP gene to the mean of 2 internal reference genes. Values are the means ± SEM, from two independent replicates spleen samples/group (n = 5). Statistical analysis was performed using one-way ANOVA followed by Fisher test. **B.** Effect of ZEA diet on matrix metalloproteinases (MMP) activity in the spleen. SDS—PAGE zymography of spleen extracts. Results are expressed as arbitrary units (A.U.). Values are the means ± SEM, from two independent replicates spleen samples/group (n = 5). Statistical analysis was performed using one-way ANOVA followed by Fisher test (* = *P*< 0.05).

The evaluation of genes expression pattern and of gelatinolytic activity of MMPs was used to complete the study of the effects of ZEA on inflammatory responses at spleen level. Unlike the increased level of pro-inflammatory cytokines expression, both MMP-2 and MMP-9 genes showed a down-regulation of their mRNA (MMP-2: 0.81 Fc, *p* = 0.654, MMP-9: 0.53 Fc, *p* = 0.413, Fig 5A.) in spleen tissues isolated from pigs fed ZEA diet. The protein activity was evaluated by gelatin-zymography, and the results presented in Fig 5B showed also a significant decrease of MMP-2 and MMP-9 activity with 17.82%, *p* = 0.001 and 2.76%, *p* = 0.010, respectively. The mRNA levels for TIMP-1 and TIMP-2, natural inhibitors of MMPs activity and secretion, were found to be slightly increased in spleen from pigs fed ZEA diet, in comparison with control samples (TIMP-1: 1.61 Fc, *p* = 0.075; TIMP-2: 1.44 Fc over control, *p* = 0.124, Fig 5A).

### Effects of ZEA diet on p38/JNK1/JNK2 MAPKs in swine spleen tissues

**Fig 6:**
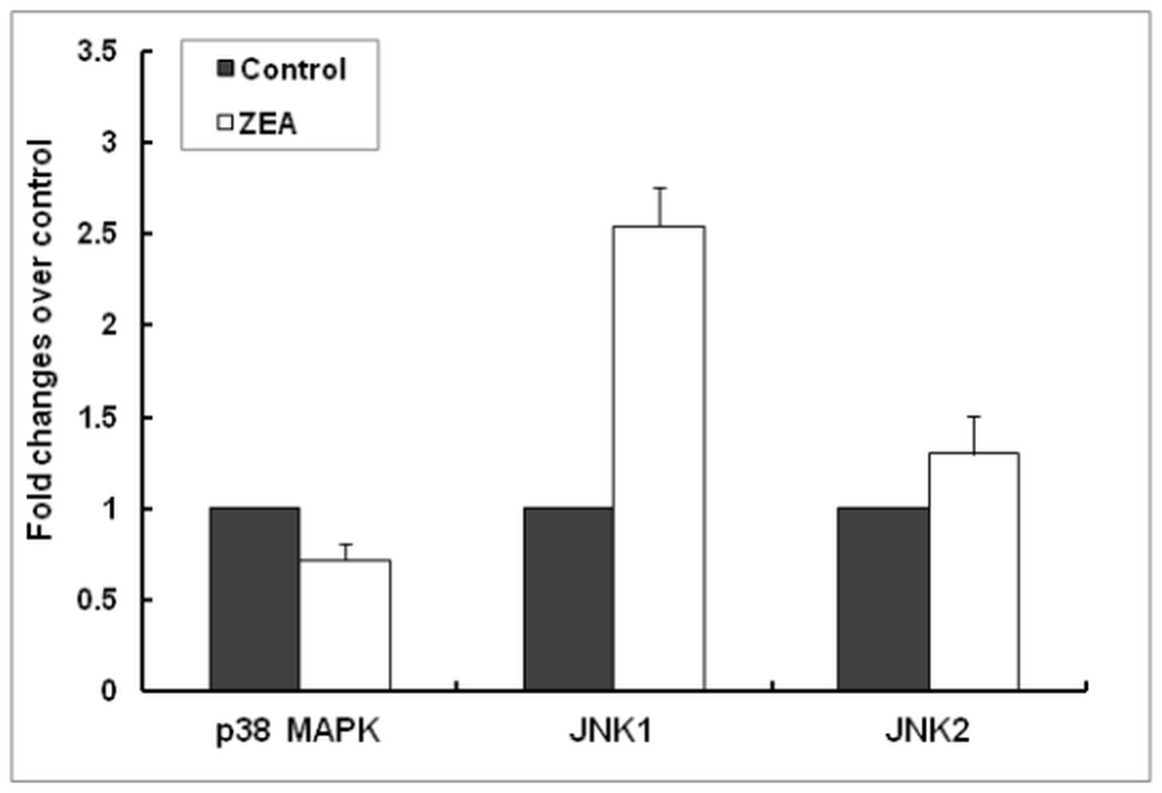
Effect of ZEA diet on mitogen-activated protein kinases (MAPKs) gene expression in spleen. Gene expression of MAPKs (p38, JNK1, JNK2) in spleen samples derived from animals treated *in vivo* with ZEA and analysed by qPCR. Results are expressed as fold changes after normalization of the expression of target gene to the mean of 2 internal reference genes. Values are the means ± SEM, from two independent replicates spleen samples/group (n = 5). Statistical analysis was performed using one-way ANOVA followed by Fisher test.

To examine the effects of ZEA diet on spleen MAPK signaling cascade, the expression of three genes belonging to this pathway (p38, JNK1 and JNK2 MAP kinases) was analyzed by qPCR. ZEA diet induced a 2.54 Fc up-regulation of JNK1 gene expression (*p* = 0.072) and a slight down-regulation of p38 MAPK mRNA (0.71Fc, *p* = 0.530). No effect on JNK2 gene expression (1.30 Fc, *p* = 0.420) was noticed (Fig 6).

**Fig 7:**
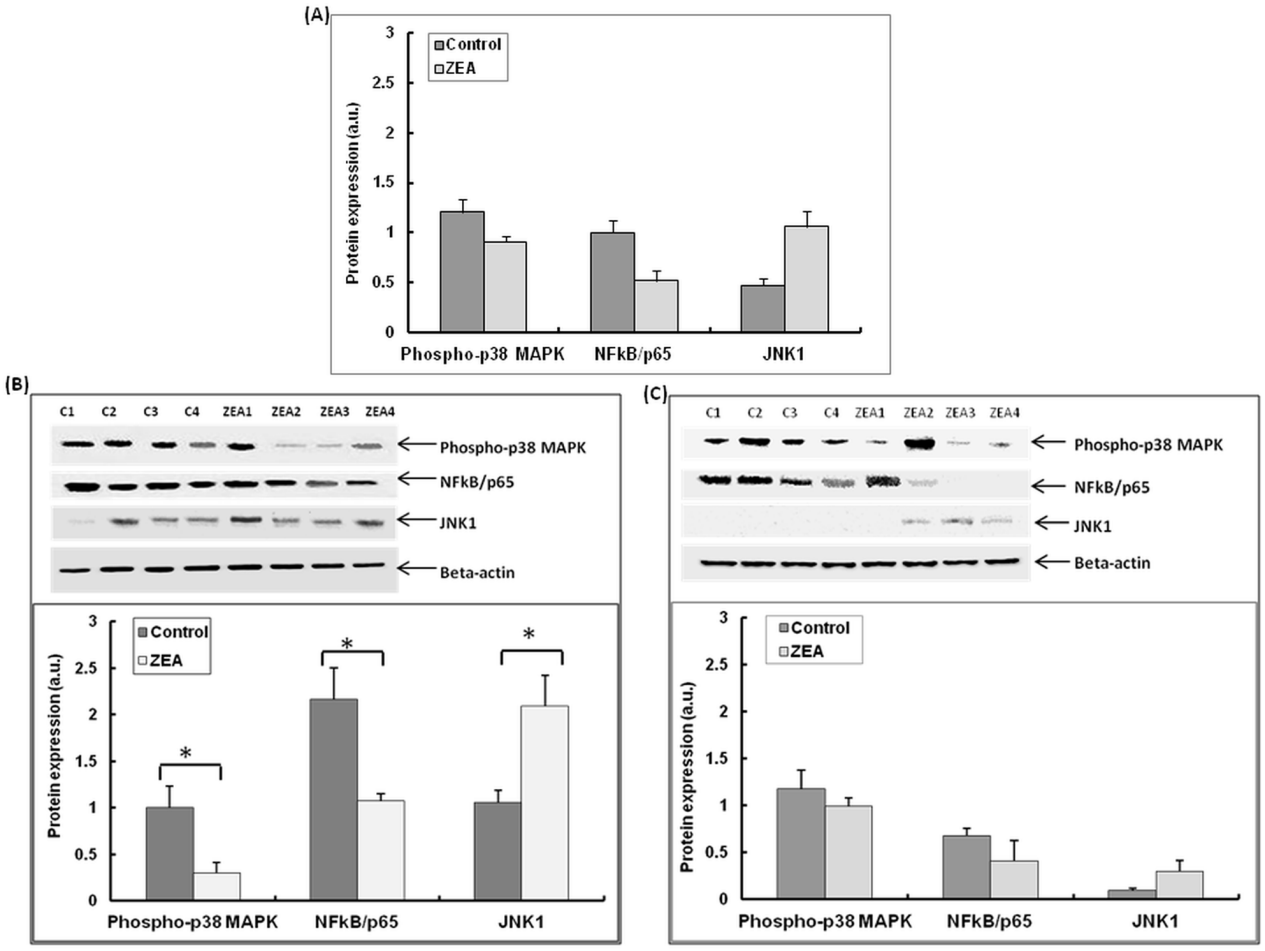
**A.** Effect of ZEA diet on the phosphorylation level of MAPKs and NF-kB in total spleen lysates. Phospho-p38 MAPK, phspho-NF-kB/p65 and JNK1 expression level determined by using Western blot analysis, and expressed as ratio of phospho-p38 MAPK, phspho-NF-kB/p65 and JNK1 to β-actin band intensities. Results are expressed as arbitrary units (A.U.). Statistical analysis was performed using one-way ANOVA followed by Fisher test (* = *P*< 0.05, ZEA-contaminated spleen versus Control spleen).**B.** Effect of ZEA diet on the phosphorylation level of MAPKs and NF-kB in cytoplasmic spleen lysates. Phospho-p38 MAPK, phspho-NF-kB/p65 and JNK1 expression level determined by using Western blot analysis, and expressed as ratio of phospho-p38 MAPK, phspho-NF-kB/p65 and JNK1 to β-actin band intensities. Results are expressed as arbitrary units (A.U.). Statistical analysis was performed using one-way ANOVA followed by Fisher test (* = *P*< 0.05, ZEA-contaminated spleen versus Control spleen). **C.** Effect of ZEA diet on the phosphorylation level of MAPKs and NF-kB in nuclear spleen lysates. Phospho-p38 MAPK, phspho-NF-kB/p65 and JNK1 expression level determined by using Western blot analysis, and expressed as ratio of phospho-p38 MAPK, phospho-NF-kB/p65 and JNK1 to β-actin band intensities. Results are expressed as arbitrary units (A.U.). Statistical analysis was performed using one-way ANOVA followed by Fisher test (* = *P*< 0.05, ZEA-contaminated spleen versus Control spleen).

The immunoblot analysis showed a good agreement with data obtained by qPCR, the JNK1 total protein level being significantly increased in spleen of animals fed ZEA diet (128.04% increase over control, *p* = 0.022), while the level of phosphorilated p38 MAPK was reduced (-24.55%, *p* = 0.100) (Fig 7A). In cytoplasmic fraction of spleen lysates a significant increase of JNK1 protein level (97.16%, *p* = 0.043) as well as a significantly reduction of phosphorylation level of p38 MAPK (-69.87%, *p* = 0.049) was observed (Fig 7B). At nuclear level, ZEA treatment was also associated with the increase of JNK1 protein level (240%, *p* = 0.062) and with the reduction of phosphorylation level of p38 MAPK (-15.69%, *p* = 0.450) (Fig 7C).

### Effects of ZEA diet on PPARγ/NFkB/AP-1/STAT3/c-JUN nuclear receptors in swine spleen tissues

**Fig 8:**
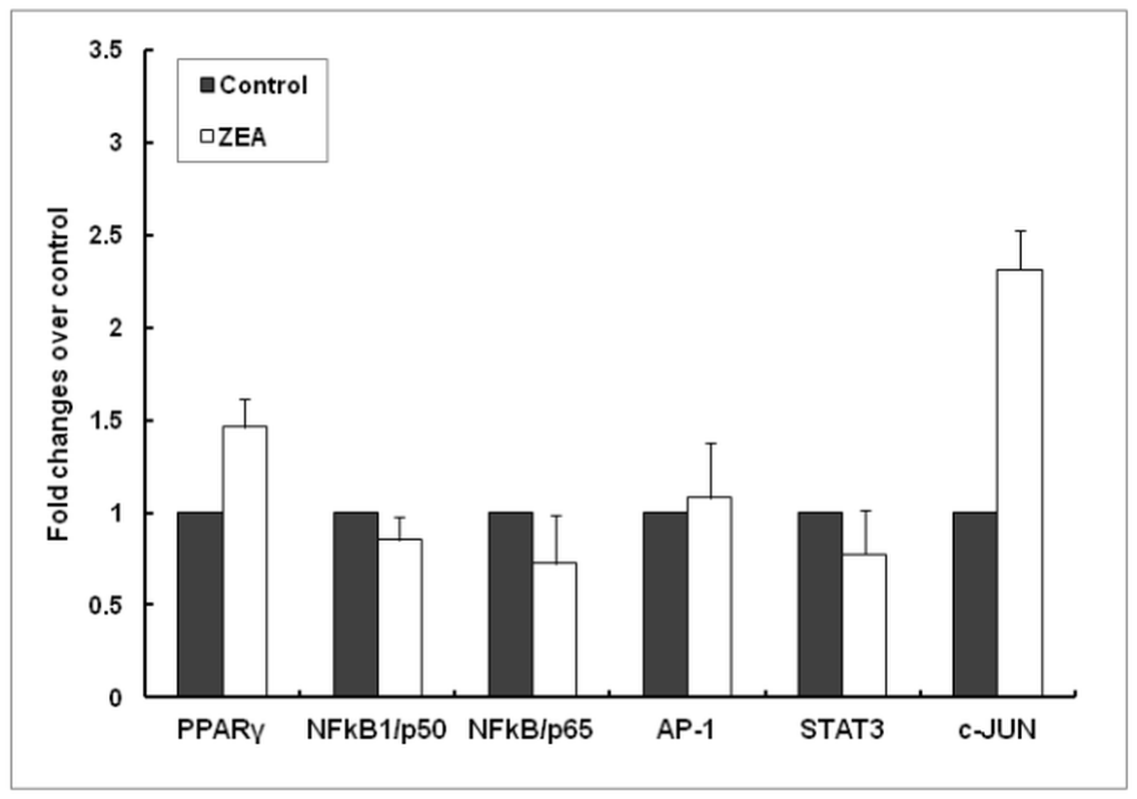
Effect of ZEA diet on nuclear receptors (PPAR-γ, NF-kB1/p50, NF-kB/p65, AP-1, STAT-3, c-JUN) gene expression in spleen. Gene expression of nuclear receptors in spleen samples derived from animals treated *in vivo* with ZEA and analysed by qPCR. Results are expressed as fold changes after normalization of the expression of target gene to the mean of 2 internal reference genes. Values are the means ± SEM, from two independent replicates spleen samples/group (n = 5). Statistical analysis was performed using one-way ANOVA followed by Fisher test.

The expression of genes coding for PPARγ, NFkB/p50, NFkB/p65, AP-1, STAT3 and c-JUN nuclear receptors were analyzed by Real Time PCR in order to understand the in-depth effect of ZEA in spleen. Our experiment demonstrated that after exposure to dietary ZEA toxin, c-JUN mRNA was increased by 2.31 Fc (*p* = 0.181) in comparison with control group whereas the expression of PPARγ and AP-1 gene expressions was slightly increased (1.46 Fc, *p* = 0.352 and 1.08 Fc, *p* = 0.796, respectively) and that of NFkB/p50 (0.85 Fc, *p* = 0.708), NFkB/p65 (0.73Fc, *p* = 0.424) and STAT3 (0.78 Fc, *p* = 0.499) slightly decreased in spleen samples isolated from piglets fed ZEA diet (Fig 8).

The immunoblot analysis showed that the phosphorylation level of NFkB (NFkB/p56) was reduced in cytoplasmic (-52.90%, *p* = 0.015) as well as in nuclear fractions (-39.27%, *p* = 0.316) in spleen samples collected from animals receiving dietary ZEA (Fig 7B and 7C). As expected, the total level of NFkB/p65 was reduced in samples from pigs fed with ZEA (-48.48%, *p* = 0.012 Fig 7A).

### Gene interaction network

**Fig 9:**
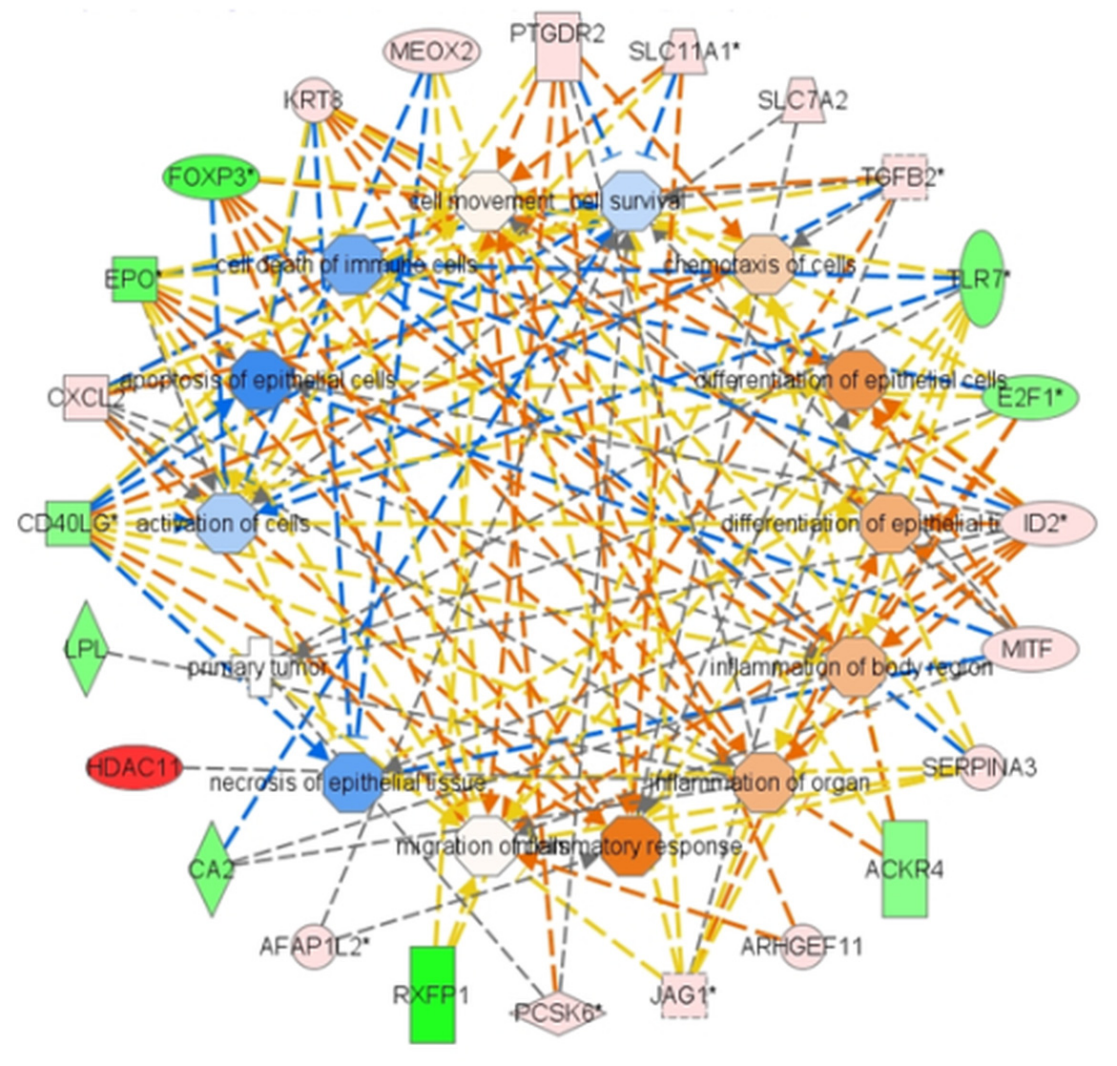
Gene Network. Predicted connections between the differentially regulated genes in spleen. Information about the regulation of genes is included in the figure: the red and green gradient colour from dark to light shows the degree of genes up-regulated or down-regulated respectively from low to high in spleen of ZEA treated pigs versus spleen of control pigs. Nodes were used to connect the regulated genes; the orange nodes means predicted activation, blue nodes means predicted inhibition. Orange and blue lines show predicted relationships leading to activation or inhibition respectively. Yellow and grey lines show inconsistent or no predicted effects.

Using Ingenuity Pathway Analysis we obtained the genes interaction network presented in Fig 9 predicting the biological processes that might be affected by ZEA toxicity in human. The data shown in Fig 9 are drawn from the pig study and provide a hypothetical basis for ZEA effects in the human system. This network including 23 differentially regulated genes (14 up-regulated and 9 down-regulated genes) and 13 connecting nodes with different degree of regulation showed that ZEA could modulate in human inflammatory processes, cellular differentiation, cellular apoptosis and cell death of immune cells. The down-regulation of FOX3, EPO, CD40LG genes, for example and the up-regulation of CXCL2, PTGDR2, KRT8, MEOX2, SLC11A1 might activate inflammatory responses, the inflammation of body regions and organs as well as the differentiation of cells. Meanwhile the down-regulation of TLR-7, EDF1, CDL40 genes expression and the up-regulation of TGFB2, SERPINA3 might inhibit the activation, apoptosis and the death of immune cells. The degree of genes up-regulation or down-regulation is reflected by the red and green colours; the colour gradient from dark to light shows the low to high amplitude of the effects of ZEA.

## Discussions

Among farm animals, pigs, particularly the gilts, are the most sensitive species to ZEA effects [13, 8]. The most common pathological effects are those affecting reproductive system, the female pigs being sensitive to estrogenic effects of ZEA and its metabolites. Using a microarray approach we first evaluated the effect of ZEA on the global transcriptome expression in spleen of pigs fed ZEA contaminated diet for 18 days. The microarray data indicated 480 genes with an altered profile by compared to the control group; of these, 288 genes were up-regulated and 192 down-regulated. Pathway analysis showed that ZEA affected genes belongs to 6 functional clusters (signalłing, cellular proliferation, transcription factors, growth factors, cytokines and inflammatory response), the highest number of genes being involved in cellular signalłing pathway (46% of total number of differentially expressed genes). Similar data were reported by Parveen et al., (2009) [43], who found that the genes related to cellular signalłing were the most numerous genes affected by ZEA treatment in MCF-7 cells. In our study, the down-regulation was the predominant effect of ZEA contaminated diet exerted on the genes related to cellular signalłing, 36 genes being down-regulated and only 21 genes up-regulated (Table 3). RXFP1 gene encoding for relaxin receptor was the most significantly down-regulated (0.25 Fc); this receptor activates a wide spectrum of signalłing pathways and induces the activation of several G protein-coupled receptors to produce important messengers like cAMP and nitric oxide [44]. Also, RXFP1 is able to activate Erk1/2 tyrosine kinase [44] an estrogenic-sensitive MAP-kinase and this could explain the effect of ZEA as estrogenic disruptor [45]. Indeed, uterine RXFP1 transcripts were decreased in piglets as a consequence of continuous ZEA exposure [45]. The most up-regulated signalłing gene was OR10J3, involved in innate immunity and associated with risk for breast cancer in women [46]. Other genes such as NXPH3 (neurexophilin3) and TNNC2 (troponin C type 2), responsible for the synthesis of neuroxophilin and troponin, proteins promoting the adhesion between dendrites and axons and regulating the striated muscle contraction were also highly up-regulated (6.50 and 9.13 Fc respectively).

In our study, ZEA treatment affected genes related to proliferation and cytokine networks, 14% and 13% of total altered genes, respectively. Microarray screening showed a significant increase in JAG1 (2.43 Fc) and TGFβ-2 (2.0 Fc) gene expression and a decrease in FoxP3 and TLR7 genes (0.31 and 0.40 Fc respectively). JAG1 gene encode for jagged 1 protein, important for its connection with Notch receptors and for the role in development of certain type of cells in the growing embryo and new blood cells. A previous study of Choi et al., (2009) [47] revealed that JAG1 siRNA induced changes in inflammatory-related genes and the study of Lemmer et al., (1999) [48] showed a progressive increase in gene expression for TGF-α and TGF-β1 in liver specimens of rats fed another *Fusarium* mycotoxin, fumonisin B1. FoxP3 is a master gene involved in immune tolerance mechanisms, and it has been suggested that mycotoxins treatment could affect FoxP3 expression. For example, treatment of porcine jejunal explants with deoxynivalenol, another toxin produced by the same fungal species [49] leaded to a strongly repression of FoxP3 gene expression correlated with a subsequent potentiation of the inflammatory response in the gut [50], while the treatment of primary porcine alveolar macrophages with T2-toxin resulted in the decrease of TLR7 [51].

10% and respectively 7% of total number of differentially expressed genes belongs to transcription and growth factors clusters. The most significantly up-regulated genes were HDAC11 (25.99 Fc, Table 3), a negative regulator of IL-10 production [52] whose up-regulation has been seen in many cancer cells. ADAM12, PCSK6 and AFAP1L2, identified as modulators of inflammation in diverse systems when their expression are up-regulated [53–55] were also significantly higher induce (2.75, 2.66 and 2.46 Fc respectively). Buske et al. (2001) [56] reported that the balanced expression of HOXA10 is crucial for the development of human haematopoiesis and in our study HOXA10 expression was among the most down-regulated transcription gene (0.19 Fc).

Spleen plays a central role in the inflammatory response as well as in the development of acquired immunity [57] and few data are available about the *in vivo* effect of zearalenone on the modulation of splenic inflammatory markers in pigs. Following the exposure to ZEA contaminated diet, 10% of the total significantly affected genes belongs to inflammatory response. Our qPCR results indicate also that ZEA increased in spleen the expression and the synthesis of pro-inflammatory cytokines (TNF-α, IL-8, IL-6, IL-1β) and had no effect on IFNγ, IL-4 and IL-10. These results are similar with those obtained by microarray analysis also (data not shown). A significant up-regulation of TNF-α (2.34 Fc), one of the most powerful inflammatory markers was observed. This over-expression might create the risk for an exacerbated inflammatory response with lesions produced at tissue level [58]. The pattern of spleen inflammatory cytokines observed in this experiment is in contrast with our previous results obtained in liver which showed a dramatically decrease for all pro-and anti-inflammatory cytokines under the effect of ZEA contamination [19]. This support the hypothesis that ZEA might have an *in vivo* biphasic effect being associated with the stimulation of inflammation in spleen and the suppression of inflammatory response in liver and that might have consequences on immune homeostasis. The increase of TNF-α under ZEA action in spleen as central player in the storage of red blood cells and lymphocytes production reveals potential cancerotoxic properties of ZEA. It was reported that over-expression of certain cytokines (TNF-α, among them) shortened the red blood cells survival, suppressed the erythroid progenitor cells and impaired iron utilization in cancer related anemia [59]. Similar effect on splenic cytokines was also observed in other farm and laboratory animal species. ZEA leaded to an increase of IL-2 and IFN-*γ* and a decrease of IL-6 gene expression in poultry stimulated splenic lymphocytes [60], while in a comparative study aiming to evaluate the effects of ZEA exposure via drinking water on immune-related parameters in different lymphoid organs, the level of IL-1, IL-10, and IFN-γ decreased in spleen of rats vaccinated or not against parvovirus vaccine and exposed to ZEA compared to those treated with vaccine alone [61].

At splenic level, ZEA altered also the morphology of splenocytes by a significant elevation of iron particle and suppressed their response to mitogen in pregnant sow fed with *Fusarium* contaminated diet [59]. It seems that spleen and the splenic cytokines response is a target for other mycotoxins action also. A significant over-expression of pro-inflammatory cytokines was observed in spleen of pigs exposed to high dose of AFB1 [62] and in spleen of mice treated with 50–100 ng/ml deoxinivalenol [63]. The activation of MMP-2 and MMP-9, other molecules involved in inflammation process as well as the increase of the MMPs inhibitor, TIMP-2 was observed in spleen of mice contaminated with T2-toxin [64]. In the present study, ZEA diet decreased significantly the MMP-2 and MMP-9 activity, down-regulated their mRNA, and slightly increased TIMP-1 and TIMP-2, natural inhibitors of MMPs. Our microarray data analysis revealed also a decrease of MMP-2 and MMP-9 mRNA (data not shown).

It was shown that ZEA exerts its effect on numerous genes expression and function through the interaction with transcription and signaling factors. After binding to estrogen receptors, the ZEA-ER complex is translocated to the nucleus where it binds to steroid-responsive elements and regulates the expression and activity of important molecules involved in cell signaling and transcription pathway [49].

Indeed in the present study the intake of dietary ZEA lead to an increase in the gene and protein expression of c-JUN kinases in both cytoplasmic and nuclear spleen lysates. JNK signaling pathway along with p-38 MAPK and NF-kB contributes to inflammatory responses in mammals [19]. The inflammatory stimulation might be a consequence of ZEA-JNK pathway activation rather than of p-38MAPK and NF-kB whose gene and protein expression were suppressed by ZEA action. This is in contrast with the significantly lower effect on pro-inflammatory response observed in liver of pigs exposed to ZEA for the same period of time which was decreased through the inhibition of p-38MAPK, NF-kB and JNK signalłing molecules [19] Inhibition of NF-κB p65 by ZEA is comparable to the effects produced by estrogen receptor ligands estradiol and estrone which are able to impair NF-κB p65 translocation into the nucleus in chondrocytes [65] and in macrophages [66]. Although there are differences between medium values of ZEA-treated animals for above mentioned parameters compared with controls, these differences were not statistically significant in all analyses; if the number of animals had been higher, perhaps these differences would have been statistically significant. Also, an increased variability in the response of animals receiving dietary ZEA in terms of IL-1β, JNK1 and c-JUN was observed. In our study, the protocol was designed to minimize the number of animals, but to adequately investigate the protocol hypothesis.

In summary, our findings showed that ZEA contaminated diet induced significant changes on global transcriptome in pig spleen. 480 genes with a significantly altered profile by compared to the control group were found through microarray analyses; of these, 60% genes were up-regulated and 40% down-regulated. Pathway analysis indicated that ZEA affected genes belonging to 6 functional clusters, 46% of total number of differentially expressed genes being involved in cellular signalłing pathway, 13% in cytokine network and 10% in the inflammatory response. Indeed, our results indicate that ZEA increased in spleen the expression and the synthesis of pro-inflammatory cytokines. The significant increase of TNF-α and HDAC11 expression reveals the potential cancerotoxic effect of ZEA. The inflammatory stimulation might be a consequence of ZEA-JNK pathway activation rather than of p-38MAPK and NF-kB whose gene and protein expression were suppressed by ZEA action. The pattern of spleen inflammatory cytokines observed in this experiment is in contrast with our previous results obtained in liver which showed a dramatically decrease for all pro-and anti-inflammatory cytokines and signaling molecules p-38MAPK and NF-kB under the effect of ZEA dietary contamination. This support the hypothesis that ZEA might have an *in vivo* biphasic effect being associated with the stimulation of inflammation in spleen and the suppression of inflammatory response in liver and that might have consequences on immune homeostasis.
